# Residual angulation of distal tibial diaphyseal fractures in children younger than ten years

**DOI:** 10.1186/s13018-014-0084-5

**Published:** 2014-10-09

**Authors:** Sung Taek Jung, Hyuk Park, Ju-Hyung Lee, Jung Ryul Kim

**Affiliations:** Department of Orthopaedics Surgery, Chonnam National University Medical School, 160, Baekseo-ro, Gwangju, 501-746 South Korea; Department of Orthopaedics Surgery, Chonbuk National University Medical School, 567 Baekje-ro, Dukjin-gu, Jeonju 561-756 South Korea; Department of Preventive Medicine, Chonbuk National University Medical School, 567 Baekje-ro, Dukjin-gu, Jeonju 561-756 South Korea

**Keywords:** Residual deformity, Distal tibia, Diaphysis, Remodeling

## Abstract

**Background:**

The purpose of this study was to evaluate the factors that influence residual angulation after treating pediatric distal tibial diaphyseal fractures.

**Methods:**

We retrospectively reviewed the records of 75 children under the age of ten who were treated at two referral centers for distal tibial diaphyseal fractures. The mean patient age was 6.8 ± 2.3 years, and the mean follow-up duration was 4.1 ± 1.3 years (range, 3 to 6 years). Early postoperative and late follow-up radiographs were used to measure angulation.

**Results:**

Twenty-four patients had valgus angulations >5° at the final follow-up. There was no varus, or anteroposterior residual angulations >5°. There was more residual valgus angulation when the postoperative angulation was >5° (*p* = 0.006) and when intramedullary nail and external fixators were applied for treatment (*p* = 0.004). Multivariate logistic regression analysis showed that postoperative angulation (adjusted odds ratio (OR) 4.33, 95% confidence interval (CI) 1.07–17.53) and treatment methods (intramedullary nail: adjusted OR 7.33, 95% CI 1.31–41.07; external fixator: adjusted OR 11.35, 95% CI 1.91–67.40 compared with the cast group) were associated with residual deformity.

**Conclusions:**

Valgus angulation after pediatric distal tibial fractures persisted in this study sample. Accurate reduction should be performed to prevent residual deformity.

## Background

The normal process of bone remodeling in the diaphysis and metaphysis of a growing child may realign initially malunited fragments. This dynamic remodeling makes the anatomic reduction less significant in a child than it is in a comparable injury in an adult. Children’s bones remodel in response to the normal stresses of body weight, muscle action, joint reaction forces, and intrinsic control mechanisms including the periosteum [1-3]. If there is any residual angulation after bone union, there is spontaneous correction. The physis responds to such malalignment by differential growth, which aligns the shaft perpendicular to the major joint reaction forces [4].

In general, axial malalignments will remodel in children after forearm or femoral shaft fractures. However, tibial angular deformities, especially distal tibial fractures, are less favorable injuries with regard to remodeling. The remodeling process in such injuries is complicated because of the activity of the muscles in the anterior and lateral compartments of the lower leg and the decreased growth potential of the distal tibia compared to that of the proximal tibia [4-6]. Cozen investigated valgus deformities after proximal tibial fractures in children [7]. In skeletally immature patients, there is femur overgrowth after mid-diaphyseal fracture; similarly, in patients under ten years old, there may be increased valgus angulation of the tibia after injury [8]. A possible cause of this valgus deformity after tibia injury is overgrowth of the tibia with tethering by the fibula.

Recently, sufficient fracture stabilization and external fixation for severe fracture can be achieved with intramedullary fixation in skeletally immature patients [9-12]. However, despite high union rates, flexible intramedullary fixation and external fixation have significant complications [13]. The most common complication observed is malunion of at least 10° in either the sagittal or coronal plane [13]. However, prior studies have not investigated residual deformities or the factors that may influence them after distal tibial diaphyseal fractures. Therefore, the purpose of this retrospective study was to evaluate residual angulation and the factors affecting residual angulation after pediatric distal tibial fractures.

## Methods

Written informed consent was obtained from the patient’s guardian/parent/next of kin for the publication of this report and any accompanying images. This study was approved by the institutional review board of Chonbuk National University, Republic of Korea. All patients under ten years old who sustained distal tibial fractures between 2001 and 2008 and were treated at one of two tertiary referral centers were included. On anteroposterior plain radiographs, the total tibial length (from the center of the tibial spine to the center of the tibial plafond) was divided into three equal parts (proximal 1/3, middle 1/3, distal 1/3). All patients with distal 1/3 closed fractures and open fractures (grades I, II, IIIA according to the system reported by Gustilo [14]) and who had been followed for more than 3 years were included in this study. Patients were excluded if they presented with undisplaced fractures, grades IIIB and IIIC open fractures, fractures associated with physeal injuries, or pathologic fractures. Patients were also excluded if there was a lack of adequate radiographs, or if they were lost to follow-up prior to 3 years after treatment.

A total of 104 fractures in 98 patients were identified, and 29 of these fractures were excluded. Of those excluded, nine fractures were undisplaced fractures, seven were Gustilo grade IIIB or IIIC fractures, three were associated with physeal injuries, and one was pathologic due to osteofibrous dysplasia. Five patients were lost to follow-up and four did not have adequate radiographs. The study includes the remaining 75 fractures from 75 patients.

Demographic data is summarized in Table 1 and includes age at the time of injury, sex, treatment method, and whether the fracture was open or closed (grades I, II, IIIA).

**Table 1:** **Patient demographics and classification**

**Parameters**		**Values**
Period		2001. 6–2008. 8
Cases		75 fracture (75 cases)
Sex (M:F)		45 (60):30 (40)
Age (years)		6.8 ± 2.3
Follow-up duration (years)		4.1 ± 1.3
Fracture configuration	Transverse	25 (33.3)
	Oblique	40 (53.3)
	Spiral	10 (13.3)
Soft tissue injury	Closed	49 (65.3)
	Open type I	15 (20.0)
	Open type II	6 (8.0)
	Open type IIIA	5 (6.6)
Fibular fracture	Intact	24 (32.0)
	Fracture	51 (68.0)
Treatment method	Cast	29 (38.7)
	Intramedullary nail	25 (33.3)
	External fixation	21 (28.0)

Data are presented as a number (percentage) or average ± standard deviation.

Radiographic data included fracture types (transverse, oblique, or spiral), presence of associated fibular fracture, and distal tibiofibular synostosis at follow-up. Radiographic angulation was measured by comparing the immediate postoperative anteroposterior and lateral radiographs to the follow-up radiographs. Residual angulation was defined as more than 5° on the most recent anteroposterior or lateral plane radiographs. All measurements were made by two independent evaluators (P.H. and L.J.H).

A senior pediatric orthopedic surgeon (S.T.J. or J.R.K.) determined the surgical method, and all patients were treated by one of these two surgeons. Closed fracture reduction was performed under general anesthesia with fluoroscopic assistance in the operating room. If the closed reduction was acceptable, a cast was placed for immobilization. If the reduction was not stable, either intramedullary nails (ESIN, Titanium Elastic Intramedullary Nail, Synthesis, USA) or external fixation (EF, AO ASIF External Fixator, Synthesis, USA) was performed. We did not perform fibular fracture fixation. All patients wore a long leg splint after ESIN for 4 weeks. Weight-bearing ability was determined on the basis of fracture stability. Patients who had ≥50% cortical contact in the transverse plane were allowed to bear weight with crutches as tolerated. During EF, fluoroscopic guidance was used to place four bicortical self-drilling fixator pins in the tibia, with two proximal and two distal to the fracture site. The EF were removed when there was radiographic evidence of bridging calluses on three of four cortices, and the patient had no pain at the fracture site when bearing weight on the external fixator. Most patients were managed with a below-the-knee plaster posterior splint and remained non-weight-bearing until the first postoperative visit at 2 weeks. The splint was then removed, and the patient was allowed to bear weight as tolerated.

### Statistical analysis

Patient demographics were summarized using means and standard deviations for continuous variables and proportions for categorical variables. The study subjects were stratified into two groups based on the presence of valgus deformity. For comparison between the two groups, we changed age to a categorical variable (greater or less than 6 years). Statistical differences between the two groups were analyzed using Fisher’s exact test. Variables with a *p* value lower than 0.2 including age and sex were retained in the final logistic regression model to determine the factor(s) related with deformity. All data were analyzed using SPSS statistics software version 20.0 (SPSS Inc., Chicago, IL, USA).

## Results

The mean patient age was 6.8 ± 2.3 years (range, 4 to 10 years) at the time of injury, with 45 boys and 30 girls included. There were more left-sided (54) than right-sided (21) fractures. The mean follow-up period was 4.1 ± 1.3 years (range, 3 to 6 years).

There were 25 transverse, 40 oblique, and 10 spiral fractures. Fifty-one fractures were associated with simultaneous fibular fractures. Twenty-six (34.6%) were open fractures, with fifteen grade I, six grade II, and five grade IIIA. Twenty-nine patients were managed with a long leg cast, 25 patients with ESIN, and 21 patients with EF.

The average time to union for all fractures was 13.7 weeks (range, 6 to 22 weeks). The average time to union for closed and open fractures was 11.5 weeks and 15.2 weeks, respectively. The average time to union was 8.8 weeks (range, 6 to 10 weeks) for the cast group, 10.9 weeks (range, 7 to 15 weeks) for the ESIN group, and 16.3 weeks (range, 8 to 22 weeks) for the EF group. There were no nonunions. Two cases (8%) were complicated by infection; however, both were resolved after repeated irrigation, debridement, and intravenous antibiotics. At the final follow-up visit, one patient with an open grade IIIA fracture had a 1.5-cm overgrowth involving the injured tibia.

**Figure 1:**
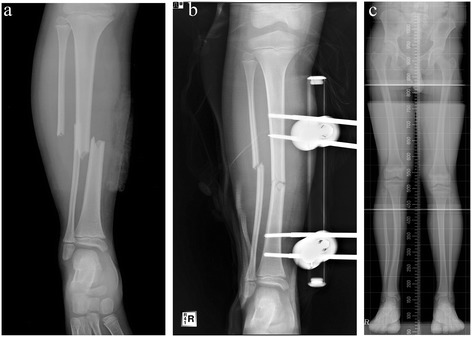
**Serials radiographs of a nine-year-old boy who sustained a right distal tibiofibular fracture. (a)** Anteroposterior radiograph at trauma. **(b)** Immediate postoperative radiograph. Treatment involved external fixation of the tibia. The postoperative angulation was 10° valgus. **(c)** A standing orthoroetgenogram after 5 years shows persistent valgus angulation.

**Figure 2:**
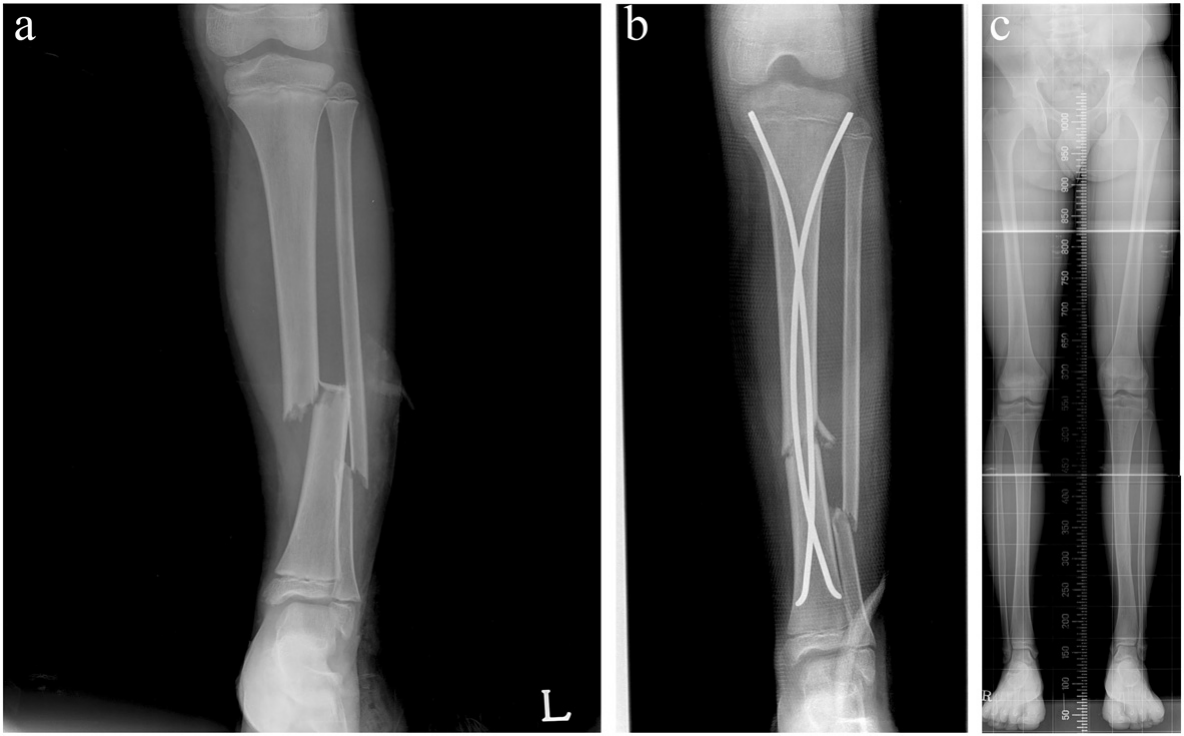
**Serials radiographs of an eight-year-old boy who sustained a left distal tibiofibular fracture. (a)** Anteroposterior radiograph at trauma. **(b)** Immediate postoperative radiograph. Treatment involved flexible intramedullary nailing. Postoperative angulation was 8° valgus. **(c)** A standing orthoroetgenogram after 5 years shows persistent valgus angulation.

**Figure 3:**
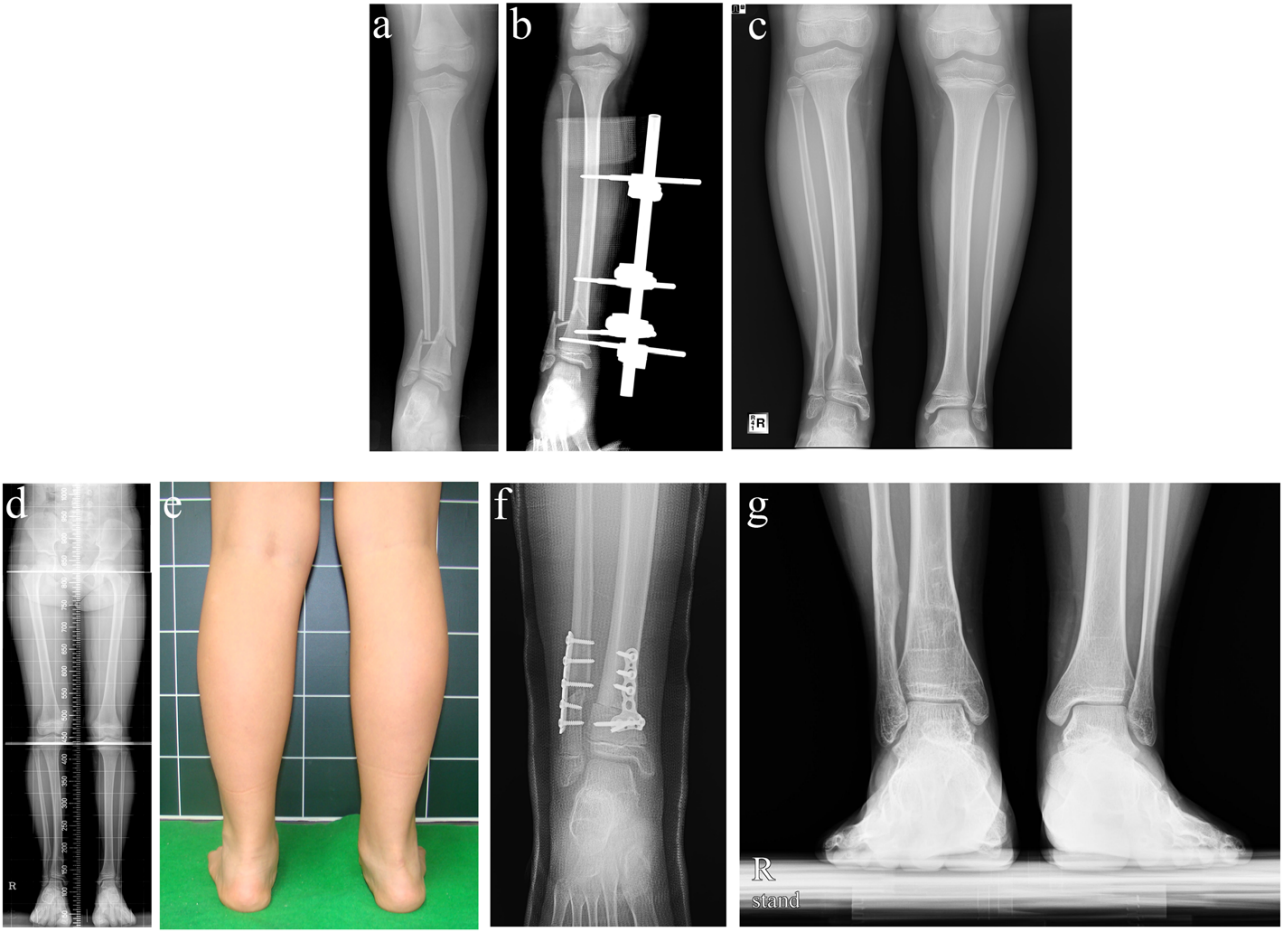
**Serials radiographs of a seven-year-old girl who sustained a left distal tibiofibular fracture. (a)** Anteroposterior radiograph at trauma. **(b)** Immediate postoperative radiograph. Treatment involved external fixation of the tibia without fibular fixation. **(c)** Standing anteroposterior radiographs after 1 year shows 20° valgus angulation. **(d)** Standing anteroposterior radiograph after 3 years of follow-up shows 15° valgus angulation and a 15-mm tibial overgrowth. **(e)** Gross image of valgus angulation around the ankle joint. **(f)** A postoperative radiograph 3 years after corrective osteotomy and shortening. **(g)** A radiograph 3 years after corrective osteotomy shows an excellent correction.

**Figure 4:**
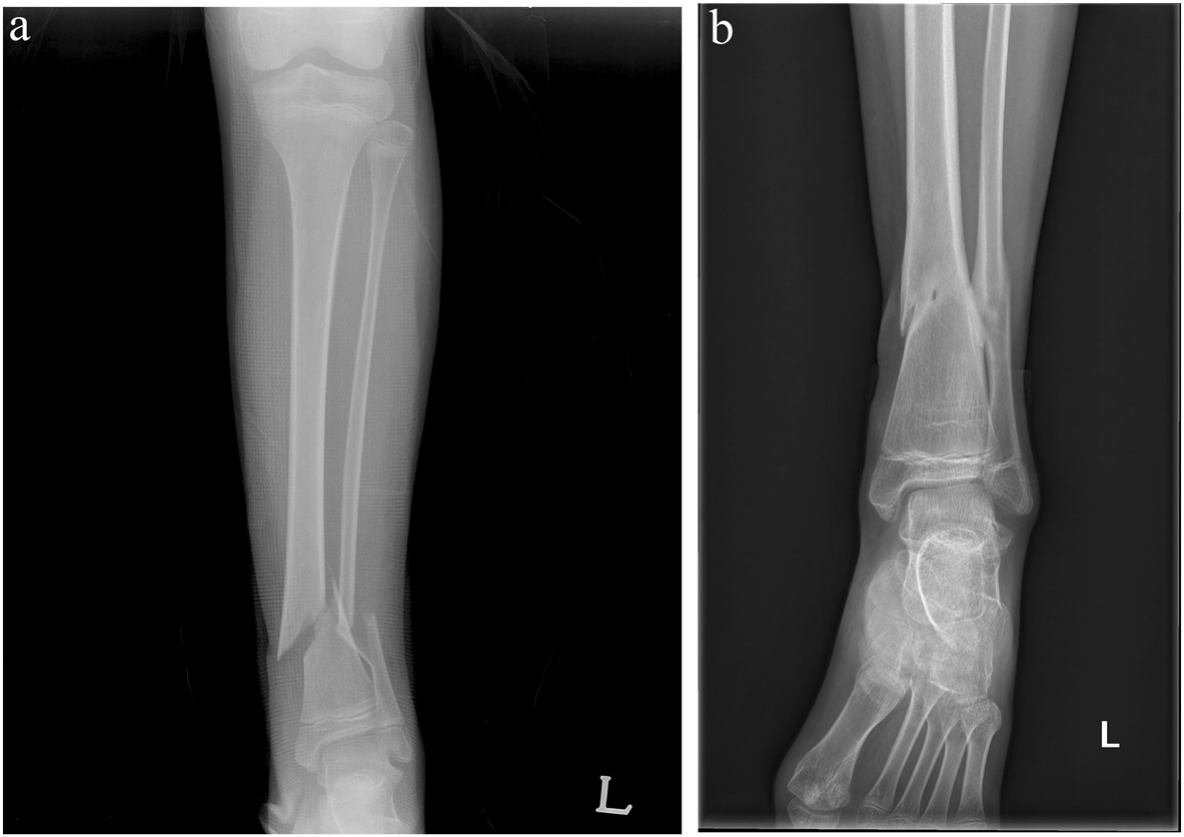
**Radiograph of a ten-year-old girl who sustained a left distal tibiofibular fracture. (a)** Anteroposterior radiograph at trauma. **(b)** A radiograph after 3 years of follow-up shows tibiofibular synostosis and an ankle valgus deformity associated with fibular shortening.

The overall improvement of postoperative angulation was 8.3° at the last follow-up visit. All residual deformities were valgus deformities (Figures 1 and 2). At the follow-up visit, 24 fractures had >5° residual valgus angulations. There were no varus anteroposterior angulations >5°. The mean changes in the valgus and varus angles immediately after surgery and at the final follow-up are shown in Table 2. In the valgus group, the mean angulation increased from the postoperative measure to that of the final follow-up. In contrast, the mean immediate postoperative angulation in varus group was shown in almost neutral angulation at final follow-up (average 0.43°, range; −0.6 to 3.9). Five patients who originally had varus angulations postoperatively had valgus angulations at the final follow-up. Another six patients who had varus angulations after surgery still had such angulations at the final follow-up. Although there was no significant difference in angulation change during follow-up between the valgus and varus groups, most of the varus angulations were remodeled and were almost neutral angulations at the final follow-up; however, most postoperative valgus angulations were either persisted or progressed. Three patients who had valgus deformities >10° after 3 years of follow-up underwent corrective osteotomy (Figure 3). Eight patients exhibited tibiofibular synostosis at the fracture site, and every patient developed valgus deformities of the ankle (Figure 4).

**Table 2:** **Angulation changes during follow-up**

	**Postoperative (°; range)**	**Last follow-up (°; range)**	**Mean change (°; range)**
Valgus group	3.49 (0.1 to 8.8)	5.47 (0.1 to 16)	2.95 (0 to 10.7)
Varus group	−3.58 (−5.9 to −2)	0.43 (−0.6 to 3.9)	4.01 (0.8 to 6.6)
*p* value			0.276

*+* valgus angle, *−* varus angle.

Clinical properties such as the radiographic results of the residual deformities are shown in Table 3 and were analyzed by Fisher’s exact test. There were no statistically significant differences in age (*p* = 0.313) or sex (*p* = 0.614) between the two groups. The fracture configuration was divided into two types (transverse vs. oblique, and spiral). There was no statistically significant difference in fracture configuration (*p* = 0.793). The subgroup analysis between oblique and spiral fracture types also did not reveal a significant difference (*p* = 0.702). Open types IIIB and IIIC fractures were excluded in this study. Thus, we compared closed fractures and open type IIIA fractures. Having an open fracture was not significantly associated with having a residual deformity (*p* = 0.797). There was no statistically significant difference in the presence of a fibular fracture (*p* = 0.793). According to the treatment methods, residual valgus deformities were observed in three (12.5%) patients in the cast group, ten (41.7%) in the ESIN group, and eleven (45.8%) in the EF group (*p* = 0.004). The Fisher’s exact test showed that the initial angulation (*p* = 0.002), treatment method (*p* = 0.004), and immediate postoperative angulation (*p* = 0.006) were all associated with having a residual deformity.

**Table 3:** **Comparisons between the normal and valgus deformity groups**

**Residual angulation**		**<5 (** ***n*** **= 51)**		**≥5 (** ***n*** **= 24)**		***p*** **value**
Age	≤6 years	22	(43.1)	7	(29.2)	0.313
	>6 years	29	(56.9)	17	(70.8)	
Sex	Male	32	(62.7)	13	(54.2)	0.614
	Female	19	(37.3)	11	(45.8)	
Fracture type	Transverse	16	(31.4)	9	(37.5)	0.793
	Oblique and spiral	35	(68.6)	15	(62.5)	
Open fracture	No	34	(66.7)	15	(62.5)	0.797
	Yes	17	(33.3)	9	(37.5)	
Fibular fracture	No	18	(35.3)	6	(25.0)	0.793
	Yes	33	(64.7)	18	(75.0)	
Operation methods	Cast	26	(51.0)	3	(12.5)	0.004^a^
	Intramedullary nail	15	(29.4)	10	(41.7)	
	External fixation	10	(19.6)	11	(45.8)	
Postoperative angulation	<5	45	(88.2)	14	(58.3)	0.006
	≥5	6	(11.8)	10	(41.7)	

Data are presented as a number (percentage) and were analyzed by Fisher’s exact test.

^a^Cast vs. intramedullary nail and external fixation.

Results of multiple logistic regression analysis are shown in Table 4. The backward addition method was used for modeling. Age and sex were included in the final model. Multiple logistic regression analysis showed that both initial angulations >5° (adjusted odds ratio (OR) 3.80, 95% confidence interval (CI) 1.12–12.87) and the treatment method compared to casting (intramedullary nail: adjusted OR 7.33, 95% CI 1.31–41.07; external fixation: adjusted OR 11.35, 95% CI 1.91–67.40) were associated with residual deformities.

**Table 4:** **Multivariate logistic regression analysis for risk factors associated with deformity**

		***N***	**Adjusted OR**	**95% CI**	***p*** **value**
Postoperative angulation	<5	59	1		
	≥5	16	4.33	1.07–17.53	0.040
Operation methods	Cast	29	1		
	Intramedullary nail	25	7.33	1.31–41.07	0.023
	External fixation	21	11.35	1.91–67.40	0.008

*OR* odds ratio, *CI* confidence interval.

The OR was adjusted for age and sex.

## Discussion

Remodeling after tibial shaft fractures is greatly influenced by the direction of angulation [15,16]. Angulation deformities are more often varus deformities than they are valgus. Regardless, remodeling of the angulated tibial shaft fracture is often incomplete [17-20]. Specifically, distal tibia fractures are less likely to remodel successfully because of the activity of the surrounding muscles in the anterior and lateral compartments of the lower leg. In addition, the distal tibia has a lower growth potential than that of the proximal tibia. Deformities to the distal tibia tend to be in the coronal plane, perpendicular to the axis of the ankle joint [6]. The development of the valgus deformity under consideration has been attributed to a growth discrepancy between the tibia and fibula, with the fibula exerting a tethering effect. There is an asymmetric stimulation of the physis when the tibia grows faster than the fibula [7]. The deformity is predominantly characterized by a lateral angulation of the tibia with the apex of the deformity at the fracture site. This anatomy implies that the tibial overgrowth results from the stimulation of its proximal growth plate; the fibula does not overgrow and therefore the deformity forms and progresses at the fracture site in the tibial metaphysis [8]. Swaan and Oppers found that in 86 children (with girls 1–8 years old and boys 1 to 10 years old) treated for tibial fractures, there was moderate spontaneous correction of the residual angulations after bone union [16]. In girls (ages 9 to 12 years) and boys (ages 11 to 12 years), approximately 50% of the angulations were corrected. No more than 25% of the deformities were corrected in children over 13 years of age. Hansen et al. reported that of 102 pediatric tibial fractures, 25 had malunions ranging from 4° to 19° [5]. At the follow-up visit, residual angular malunions ranged from 3° to 19°. Every patient had a complete correction. Spontaneous correction accounted for approximately 13.5% of the total deformity. Benneck and Steinert reported that 26 of 28 children with varus or valgus deformities at union had significant residual angular deformities at follow-up [21]. Although fractures with varus malalignments (of 5° to 13°) completely corrected at the physis, most valgus deformities (of 5° to 7°) were not fully corrected [22]. In the present study, 24 of 75 fractures in children under the age of ten were uncorrected. All residual deformities were valgus deformities. Valgus deformities require more precise reduction because they are perpendicular to the axis of the ankle joint.

During the first 18 months after a fracture in a child, there may be spontaneous tibial remodeling [6]. The remodeling capacity is determined by the child’s age and the degree of angulation [5,17]. In this study, there were no statistically significant age differences. However, every child has a different growth rate, which makes it difficult to analyze the relationship between age and angular deformity.

Severely displaced fractures and factures that were unstable after closed reduction were treated with an intramedullary nail or external fixator. Residual valgus deformities were significantly prevalent in patients treated with an intramedullary nail and external fixator. We suspected that there are two reasons that the surgical groups had a higher prevalence of residual valgus deformities. First, the postoperative angulations persisted until the final follow-up visit. Second, we did not fix fibular fractures. Although there were accurate postoperative reductions achieved with surgery, the residual valgus angulations developed during the follow-up period. In the present study, valgus angulations progressed over the follow-up period, while varus angulations improved. We suggest that a small amount of varus reduction may be better than is an acceptable valgus range. Further research is needed to determine whether the valgus deformity progression can be prevented by stabilizing the fibular fracture and maintaining the fibular length.

Posttraumatic tibiofibular synostosis is a rare complication of pediatric distal tibiofibular fractures [23,24]. Tibial tethering was noted in every patient. The normal growth pattern of distal fibular migration relative to the tibia was reversed. This reversed growth pattern resulted in a shorter distance between the proximal tibial physis and the fibula. Additionally, there was proximal migration of the distal fibular physis relative to the distal tibia. Shortening of the lateral malleolus leads to greater valgus alignment of the ankle [15,24,25]. In this study, eight patients who had tibiofibular synostosis in comminuted fractures of the distal tibia and fibula associated with the interosseous membrane also developed a valgus ankle deformity. In patients with tibiofibular synostosis, the valgus deformity of the distal tibia persisted throughout the entire follow-up period.

This study has several limitations to consider. First, we retrospectively analyzed a relatively small sample of patients over a short follow-up period. Second, the treatment method was dependent upon various clinical findings. Therefore, the treatment method may not have been an independent predictor of valgus deformity, but may have represented patients’ overall health.

## Conclusions

Spontaneous bone remodeling after distal tibial diaphyseal fractures in children is often incomplete. The goal of treating these fractures is to obtain accurate reduction and therefore prevent persistent deformities.
